# Effects of Mindful Self-Compassion on Psychological Well-Being in Psychiatric Rehabilitation: A Randomized-Controlled Trial

**DOI:** 10.1192/j.eurpsy.2022.632

**Published:** 2022-09-01

**Authors:** A. Andorfer, M. Hiebler-Ragger, P. Kaufmann, E. Pollheimer, L. Gaiswinkler, H.-F. Unterrainer, H.-P. Kapfhammer, A. Kresse

**Affiliations:** 1 Medical University Graz, Department Of Psychiatry And Psychotherapeutic Medicine, Graz, Austria; 2 Grüner Kreis Society, Center For Integrative Addiction Research (ciar), Vienna, Austria; 3 pro mente Reha, Sonnenpark Neusiedlersee, Rust, Austria; 4 pro mente Reha, Apr Graz, Graz, Austria; 5 University of Vienna, Institute For Religious Studies, Vienna, Austria; 6 Medical University Graz, Institute For Pathophysiology Und Immunology, Graz, Austria

**Keywords:** Mindfulness, self-compassion, Randomized Controlled Trial, psychological well-being

## Abstract

**Introduction:**

The evidence for the positive effects of mindfulness-based interventions on psychological well-being and physical health has been convincing in recent years. As a specific form of such an intervention, the Mindful Self-Compassion (MSC) training program was developed to promote self-compassion and mindfulness. An initial study on an adapted version of the MSC training program considered it to be beneficial in psychiatric inpatient rehabilitation.

**Objectives:**

The present study aims to further evaluate the link between MSC and psychological symptoms as well as quality of life.

**Methods:**

A randomized controlled trial was conducted from September 2020 to August 2021. A total of 228 patients (64% female, 36% male) participated in a six-week psychiatric rehabilitation program to assess the impact of an adapted MSC training program compared to the control intervention of Progressive Muscle Relaxation training (PMR) on psychological well-being. Both training programs took place once a week for 75 minutes as part of a standardized inpatient rehabilitation program. The participants completed the Self-Compassion Scale (SCS), the Brief Symptom Inventory (BSI-18), and the Short-Form-Health-Survey-12 (SF-12) pre and post intervention.

**Results:**

At the moment, statistical analyses are being carried out. Detailed results will be presented on the poster.

**Conclusions:**

The results of this study will contribute to rehabilitation research as they provide further insight into the role of MSC in the treatment of mental disorders. In addition, the clinical implications, and possible effects of changes in the rehabilitation program during the COVID-19 pandemic on the protocol and the results of this study will be discussed.

**Disclosure:**

No significant relationships.

